# Specificity Switching Pathways in Thermal and Mass Evaporation of Multicomponent Hydrocarbon Droplets: A Mesoscopic Observation

**DOI:** 10.1038/s41598-017-05160-z

**Published:** 2017-07-10

**Authors:** Rasoul Nasiri, Kai H. Luo

**Affiliations:** Department of Mechanical Engineering, University College London, Torrington Place, London, WC1E 7JE UK

## Abstract

For well over one century, the Hertz–Knudsen equation has established the relationship between thermal – mass transfer coefficients through a liquid – vapour interface and evaporation rate. These coefficients, however, have been often separately estimated for one-component equilibrium systems and their simultaneous influences on evaporation rate of fuel droplets in multicomponent systems have yet to be investigated at the atomic level. Here we first apply atomistic simulation techniques and quantum/statistical mechanics methods to understand how thermal and mass evaporation effects are controlled kinetically/thermodynamically. We then present a new development of a hybrid method of quantum transition state theory/improved kinetic gas theory, for multicomponent hydrocarbon systems to investigate how concerted-distinct conformational changes of hydrocarbons at the interface affect the evaporation rate. The results of this work provide an important physical concept in fundamental understanding of atomistic pathways in topological interface transitions of chain molecules, resolving an open problem in kinetics of fuel droplets evaporation.

## Introduction

Evaporation is a delicate and sophisticated process spanning scales from nano- to macro-scales found in nature and numerous technological applications^1–5^. For well over a century, the Hertz-Knudsen^6, 7^ relation has been used by many researchers to model the evaporation process based on a relationship between an evaporation coefficient and an evaporation rate. The evaporation/condensation coefficient (β) can be formulated depending on a type of kinetic boundary conditions, i.e., thermal (β_T_) or mass (β_m_) evaporation/condensation coefficient^8^. Numerous computational and theoretical models have given estimate of mass evaporation/condensation coefficient^9–11^ and evaporation rate (γ)^9, 12–16^ of hydrocarbon molecules based on the transition state theory (TST)^9, 10^, molecular dynamics (MD) simulations^10, 11^, molecular theory of solvation^9, 12–16^ and kinetic gas theory (KGT)^9, 13–16^. In most studies it is assumed that interfacial flows are thermally equilibrated with other phases. However, interfacial temperature discontinuity has been known since 1970s^17–21^ and has only been discussed in detail for simple fluids^22–27^. Ward and his colleagues developed statistical rate theory (SRT) based on quantum and statistical mechanics to describe interface transport properties such as mass evaporation coefficient. SRT has been successfully applied for evaporation process of simple fluids such as water and ethanol. In a very recent review^28^, Persad and Ward explicitly write; “there is a need to correctly extend the SRT to molecules for which not all molecular phonon frequencies, *ω*_i_, are known”. They then present the thermal energy dominant (TED) method, a simplified version of SRT in which all the vibrational modes are ignored by applying an approximation of thermal energy (KT) ≫ vibrational energy (ℏ*ω*_*i*_). Kapoor and Elliott also relied on the same assumption^27^. Although TED-SRT has been proposed to be “complete, functional and physically accurate”^28^ for water and methanol fluids, it does not take into account the conformational changes at the interface for complex fluids with multi conformations^18, 28^.

Motivated by conflicting results reported in refs 9–11 and in ref. 14 on modeling of interfacial phenomena in chain molecular systems, a question was raised regarding how translational modes as reaction coordinates^10^ can model transient “internal rotations” during phase transition as introduced in ref. 14. The general expression developed in ref. 10 and applied in ref. 9 for estimation of β_m_ is suggested to be valid if “*isotropic*” requirements at the interface are established^10^. In the vicinity of *n*-dodecane droplets surfaces the “*anisotropic*” effects have been suggested using ab initio molecular dynamics (AIMD) simulation^15^ and dynamic reaction coordinate (DRC) analysis, however^16^. Classical MD simulation results were consistent with a general expression for estimation of β_m_^10^ but reliability of GAFF^10^ and OPLS force field^11^ becomes particularly questionable at “interface” for molecules with multi-conformers. Support for this is to simulate surface tensions of organic molecules which have been calculated using GAFF and OPLS force fields within 10–20% of experimental values at room temperature^29, 30^. Additionally, the aforementioned non-reactive FFs^10, 11^ and NERD force field^31^ have predicted *n*-alkanes molecular orientation along the surface differently which are not in agreement with experimental measurement by vibrational sum frequency spectroscopy (VSFS)^32^. Therefore, this general expression for estimation of evaporation coefficient^10^ is likely to fail for modeling conformational changes at the interfacial layers^14^.

In this article, thermal (β_T_) and mass evaporation (β_m_) coefficients and evaporation rate (γ) are respectively calculated using novel transient reactive molecular dynamics simulations, the statistical associating fluid theory (SAFT) and “quantum transition state theory/improved kinetic gas theory” (QTST/IKGT)^14^. We apply a non-equilibrium MD simulation technique using ReaxFF^33^ and SAFT equation of state^34^ with quantum chemical calculations^35^ to fundamentally understand how interfacial flows in *n*-dodecane droplets affect β_T_ and β_m_ kinetically/thermodynamically. We also develop and present the results of a new version of QTST/IKGT for multicomponent hydrocarbon systems which have been inspired by the “*discrete*” Boltzmann method. These findings provide an important physical concept: dynamic coupling between liquid and gas phases during the evaporation, which should be useful in better understanding the simultaneous influences of thermal and mass transfer on the evaporation rate of multicomponent fuel droplets at the atomic level.

## Results and Discussion

A non-equilibrium MD simulation technique using ReaxFF is proposed to determine thermal evaporation coefficient of *n*-dodecane, a representative of *n*-alkanes in Diesel fuel. The reliability of reactive force field ReaxFF has also been assessed and compared for modeling the evaporation of hydrocarbons^15^ using the quantum chemical calculations (DFT and PM7 methods) and available experimental data on the determination of bond energies, Gibbs free energies of internal molecular dynamics of a set of *n*-dodecane conformers and collision energies of attacking molecules with the surface of the droplet. It was shown that ReaxFF performs better than semi-empirical quantum chemistry PM7 method in terms of both cost and accuracy of calculations of the evaporation of *n*-dodecane. Therefore, the bond energy bond order approach of ReaxFF is applied to study thermal effects induced over the interfacial flows during the evaporation process of *n*-dodecane. The aim of this simulation is to investigate whether the thermal coefficient values are temperature dependent similar to mass evaporation/condensation coefficient, while we examine the interfacial temperature discontinuities. The thermal evaporation coefficient is defined as:

1 $${\beta }_{T}=\frac{ < {T}_{i}-{T}_{g} > }{ < {T}_{l}-{T}_{g} > },$$

where *T*_i_ *, T*_*g*_ and *T*_*l*_ refer to, respectively, the effective temperatures in the interfacial layer, gas and liquid phases assuming a semi-spherical droplet evaporates into vacuum without any movement (see Methods). The results of this simulation will give us important information for better understanding the energy transfer mechanisms in the initial transient stage of the evaporation process. The vacuum conditions have already been investigated experimentally, theoretically and computationally on simple fluids^36–39^.

**Figure 1:**
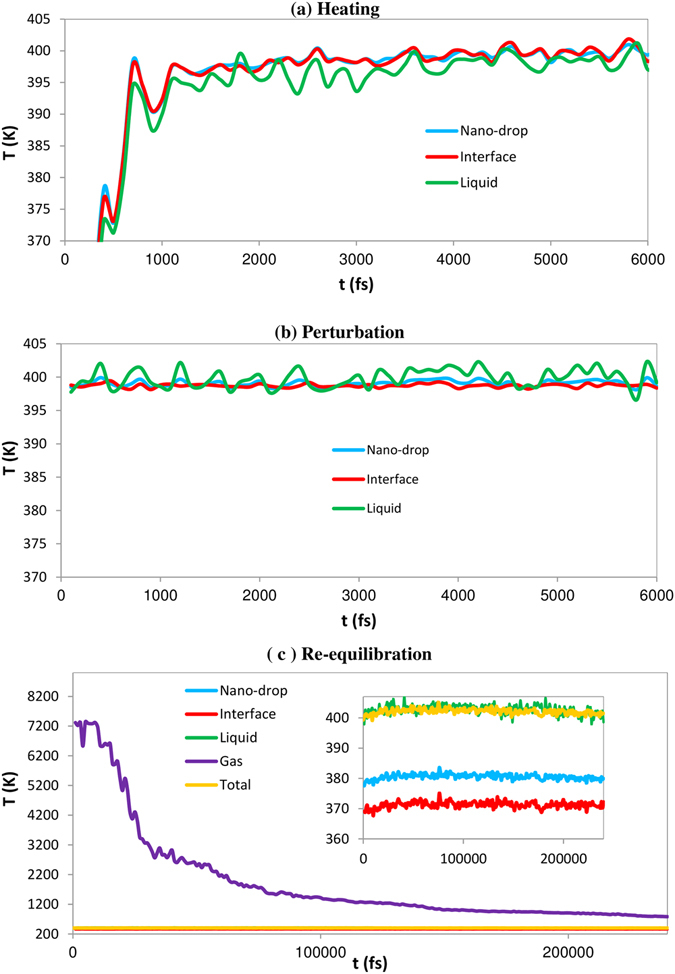
Temperatures at the surface of a nano-droplet. (**a**, heating, **b**) perturbation and (**c**), re-equilibration stages are presented at initial temperature 400 K. The temperature jump is observed at the interface during the re-equilibration stages due to cooling effects.

**Figure 2:**
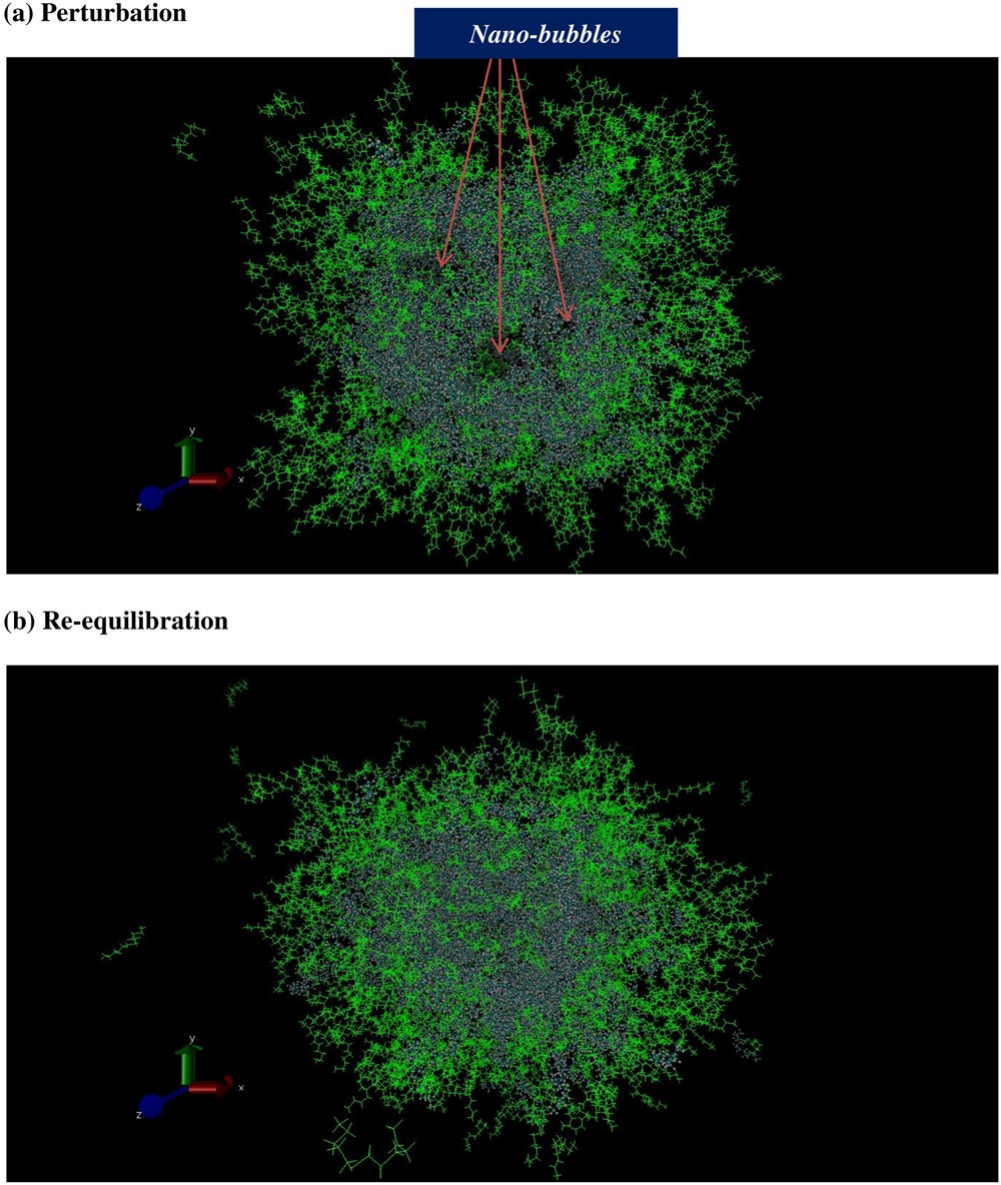
Snapshots of a nano-droplet during the evaporation process. (**a**) Formation of nano-bubbles in liquid phase after 6 ps perturbation produced by inverted heat energy pumping from gas phase (top image). (**b**) Disappearance of nano-bubbles after 50 ps re-equilibration (bottom image).

The time evolutions of various average molecular energies and corresponding temperatures were obtained at various stages of droplet heating and evaporation, identified as *heating*, *perturbation*, and *re-equilibration*. The values of temperatures are shown in Fig. 1. The initial droplet heating was set up to take place during 1000 fs using the Berendsen’s thermostat^40^. During this period the average temperature of the droplet reached 400 K. At the later times some oscillations of droplet temperature were observed, with the liquid temperatures being almost always below the interface temperature. This stage is called the *heating* stage (see Fig. 1a). At the next stage, the system was *perturbed* using various coupling time constants (τ_T_) as described in Methods. When the interface is strongly coupled to the thermostat using τ_T_ = 1 fs and *T*_*0*_ = 400 K, the temperature is controlled by the velocity rescaling algorithm used in the Berendsen’s thermostat. But when τ_T_ = 100 ps is specified for liquid phase, the temperature is maintained only through thermal effects induced by conformational changes since the thermostat does not function due to very slow rate of change of kinetic energy, therefore canonical ensemble (NVT) is essentially converted to the micro-canonical one (NVE). Application of these two different coupling time constants on a liquid drop leads to the control of liquid and interface temperatures in two different ways causing an oscillation of liquid phase temperature around the droplet temperature and showing transient transfer of energy mostly between liquid and interface since internal rotations or torsions do not change the centre of mass of molecules (see Fig. 1b). The averaged liquid temperature value was determined to be higher than interface one during the *perturbation* stage (*T*_l_ = 399.83 K against to *T*_i_ = 398.72 K). This discrepancy can be explained by the fact that molecules at the surface with high energy leave the drop leading to cooling effect caused at the interface. Moreover, as already mentioned our analysis in estimation of temperature has been done on molecules that stay in their relevant sub-systems (liquid or interface) during the evaporation process. After imposing these non-equilibrium conditions, the formation of some nano-bubbles of 1–3 nm in diameter was observed in the liquid phase. This illustrates how inversion of heat energy affects the structure of the liquid phase. These nano-bubbly flows into the droplet gradually disappeared when the sub-systems (gas, interface and liquid) reached the quasi-equilibrium state and molecules in the liquid phase could show expected behaviours again (compare structures in Fig. 2 after stages of b and c). As seen in Fig. 1c, the liquid phase has temperature higher than the droplet and even the interface during the re-equilibration in which system will be simulated using coupling τ_T_ = 100 ps implying minimal perturbation effects caused by thermostat. This is related to the fact that the directions of transfer of heat and mass are not the same during evaporation leading to higher temperatures in the liquid phase relative to the interface. Gas temperature during the evaporation drops about an order of magnitude and reaches the saturation state as the energy transfers from the gas phase to the interface and then into the liquid phase in a stepwise manner. As shown in Table 1, values of the evaporation coefficient are identical at temperatures 350 and 400 K with a time constant of 2.3 × 10^−4^ ps^−1^ and we can expect that those do not change dramatically at higher temperatures as well.

**Table 1:** Values of temperatures and thermal evaporation coefficients.

Potential	Model	T_total_	T_gas_	T_Liquid_	T_Interface_	T_nano-drop_	*β*_*T*_
ReaxFF	3-D	402.10	~1923.5	402.74	371.27	380.54	0.98
		352.37	~872.8	352.65	321.35	330.47	0.98

Thermal evaporation coefficient (*β*_*T*_) at temperatures of 350 and 400K—while simulations in literature have been performed at constant temperatures to estimate mass evaporation coefficient *β*_*m*_ in an one dimensional model^9–11^, temperature gradient at interface could be modelled using the ReaxFF method in a three dimensional model.

Mass evaporation coefficient can be derived in terms of thermodynamic potentials and SAFT molecular based equation of state^34^. SAFT can be applied for predicting interfacial layer thickness of fluids and it incorporates the effects of chain length, molecular association and other interactions such as long-range dipolar forces and dispersions. While the interfacial layer effects were not explicitly modelled in refs 9, 13, 15 and 16, we consider these effects in this study by setting up an equation including interfacial width, δ. A standard state has to be defined for the evaporation/condensation process and with this thickness the relationship between the free energy of evaporation/condensation (ΔG_g↔int_) and the coefficient β_m_ becomes:

2 $$\frac{(\frac{\langle {\beta }_{m}\rangle }{\delta })}{1-\langle {\beta }_{m}\rangle }=\exp (-\,\frac{\langle {\rm{\Delta }}{G}_{g\leftrightarrow \mathrm{int}}\rangle }{RT}),$$

where <Δ*G*_g↔int_> presents the average difference values of Gibbs free energy of conformers in the interfacial layer and gas phase (see Methods). Taking the interfacial layer effects and relevant correction terms into consideration, the same results are obtained as reported in ref. 9. One question arises concerning whether or not adding the interfacial layer using SAFT has had no effects on evaporation/condensation coefficient of *n*-dodecane. The answer is no since SAFT, which is a thermodynamic-based approach, cannot model properly transient processes such as the internal rotations in chain molecules^14^. While this molecular theory can provide useful interfacial properties, it cannot describe the interface at an atomic level. More specifically, in all diffuse interface models the existence of interfacial width is inherent and once it reduces to a length scale which is small in comparison with the macroscopic length scale associated with the motion of the two bulk fluids, these models are related to the free-boundary problems^41^. We believe that these sorts of equations are fundamentally unable to track “thermal effects” induced with “transition states” over the interfacial layers during the evaporation process^42^. We do not think that classical diffuse interface models can capture “quasi-equilibrium” transition states and internal molecular dynamics effects in complex molecules which have multi-structural effects. The internal rotations in multi-conformers cannot be modelled based on classical and harmonic models and therefore anharmonicity effects (conformational changes and the coupling between torsions and vibrational modes) should be considered based on quantum mechanics theory and a suitable statistical mechanics method in which the atoms in molecules (AIM) motions are taken into account.

In order to understand simultaneous relationship between thermal and mass evaporation with evaporation rate in multicomponent fuel droplet hydrocarbons, we have applied an extension of the quantum transition state theory/improved kinetic gas theory (QTST/IKGT)^14^. The evaporation flux is first predicted based on the assumption that single molecular events occurring during the evaporation of individual components from a multicomponent liquid phase are independently and identically distributed; and then we generalize the total solution evaporation flux for a *c*-component system as a summation of individual component evaporation fluxes which are in equilibrium in gas and liquid phases. These expressions can be easily applied to mixtures with any number of chemical components (and not just binary mixtures, as is the case for this study because of the available experimental data):

3 $$J=\sum _{i,\,jk=1}^{n}\sum _{C=1}^{m}{J}_{i,jk}^{C},$$

where

4 $${J}_{i,jk}^{C}=({[\frac{2\pi {k}_{i}^{C}}{{\omega }_{i}}]}^{TST}\times {[\frac{{k}_{jk}^{C}}{{A}_{jk}}]}^{KGT}),$$

where

5 $${k}_{i}^{C}({\rm{TST}})=\frac{(\frac{1}{{k}_{B}T})}{(\frac{1}{{k}_{B}T}-\frac{1}{{\alpha }_{i}})}[{\exp }^{(\frac{1}{{k}_{B}T}-{\alpha }_{i}){U}_{i}^{{[{R}_{i}-{P}_{i}]}^{\#}}}-1]\,\frac{{k}_{B}T}{h}\,\exp \,(-\frac{{G}_{i}^{{[{R}_{i}-{P}_{i}]}^{\#}}}{{k}_{B}T}),$$

and

6 $${k}_{jk}^{C}({\rm{KGT}})={(\frac{1}{{m}_{j}}+\frac{1}{{m}_{k}})}^{0.5}{({r}_{j}+{r}_{k})}^{2}{(8\pi {k}_{B}T)}^{0.5}(\frac{p}{{n}_{0}{k}_{B}T})\,\exp \,(\frac{\langle {\rm{\Delta }}{G}_{g\leftrightarrow l}^{C}\rangle }{RT})$$

where C indicates the number of chemical components in the system, j is the index corresponding to gas/vapour molecules or clusters/droplets colliding with conformers i at the surface of other clusters/droplets and gas/vapour molecules with the index k. *α*_*i*_ = 1/(*ħω*_*i*_) in that ω_i_ refers to magnitude of transition-state imaginary frequency of conformers i and A_jk_ represents the gas/vapour molecules or clusters/droplets accessible surface area. $\langle {\rm{\Delta }}{G}_{g\leftrightarrow l}^{c}\rangle$ presents the average difference values of Gibbs free energy of each component between liquid and gas phases. ${G}_{i}^{{[{R}_{i}-{P}_{i}]}^{\#}}$ is the activation Gibbs free energy induced by internal rotations in each conformer including zero-point energy. The m_j_ and r_j_ present the mass and radii of gas/vapor molecules or clusters/droplets colliding with other clusters/droplets and gas/vapour molecules with the mass m_k_ and radii of r_k_.

**Figure 3:**
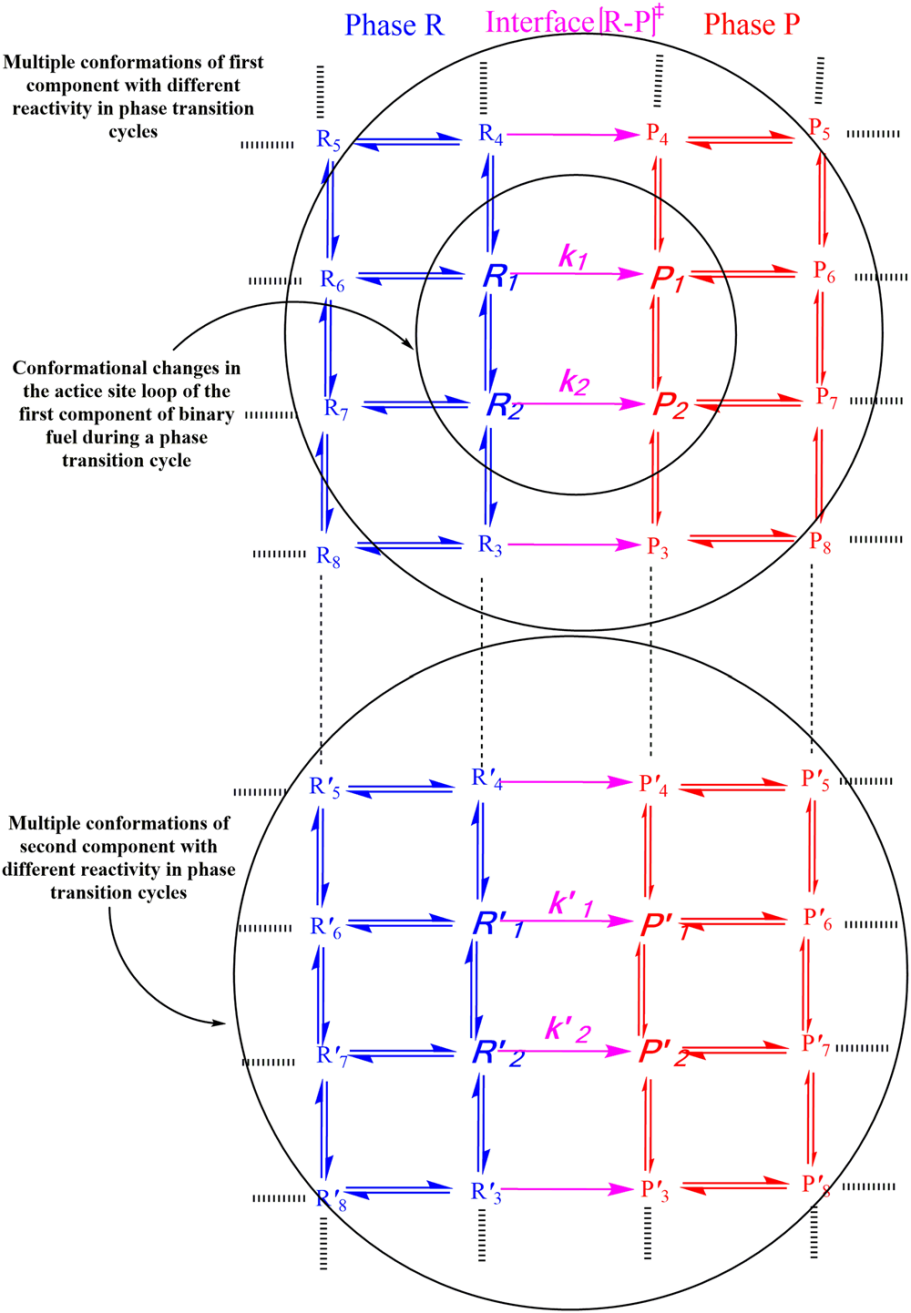
A *n*-states schematic of QTST/IKGT in which two conformational states are actively involved in phase transitions at the vicinity of a binary fuel-Two conformers depicted by R_i_ (Rʹ_i_) and P_i_ (Pʹ_i_) in R and P phases are in equilibrium state with each other and in quasi-equilibrium state with some transition states at interface [R_i_ − P_i_]^≠^. Two-state hybrid kinetic model used to fit experimental kinetic data for a mixture of *n*-heptane and *n*-hexadecane molecules.

**Figure 4:**
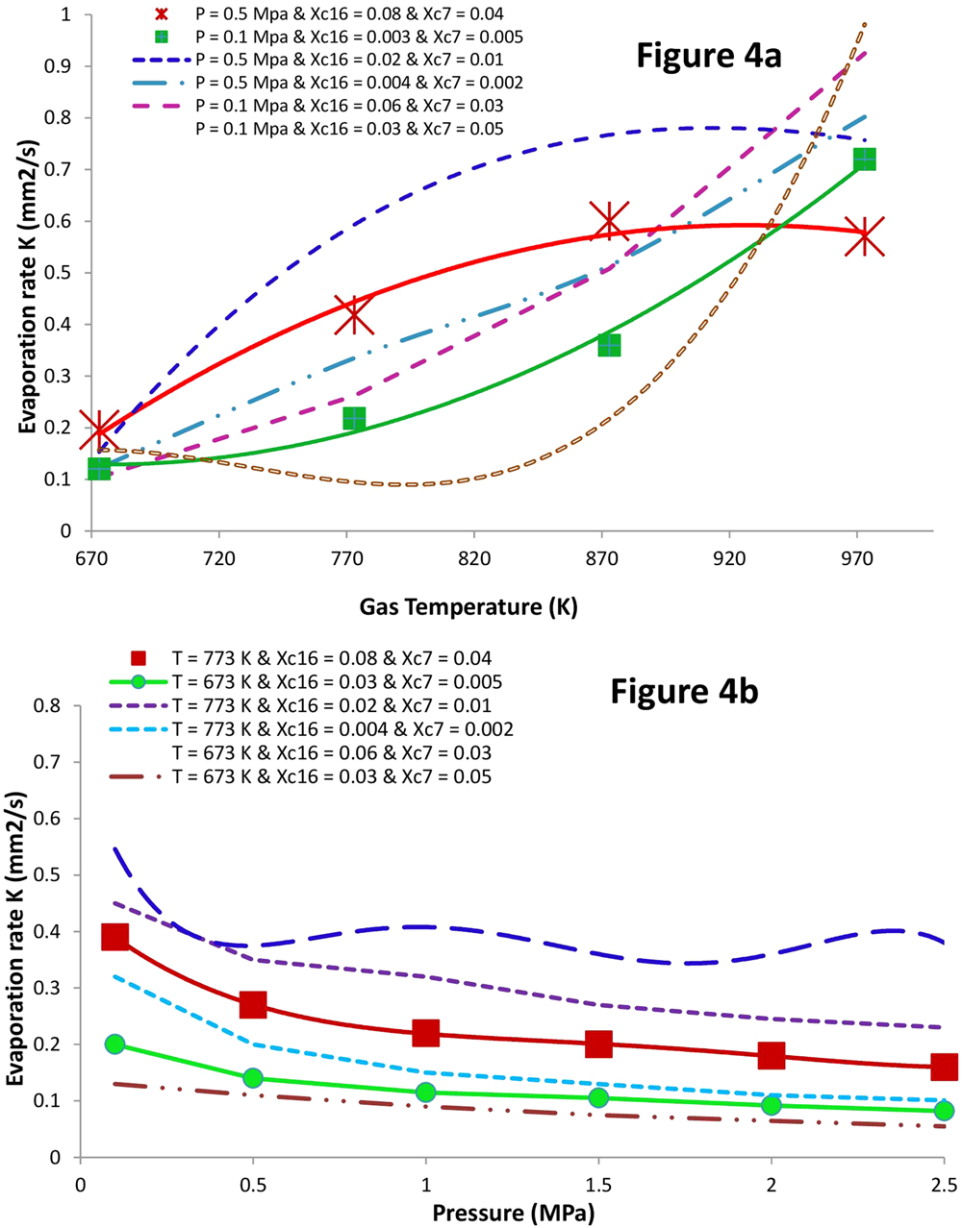
Evaporation rates of a binary fuel droplet. The fits show that QTST/IKGT reproduces temperature- and pressure-dependent evaporation rate in binary fuel droplet with 1.2 mm diameter. The (un) circles and solid (dash) lines respectively represent experimental measurements and results obtained by our model — with the parameters given in Table 2. The fitted data present effects of (**a**) temperature and (**b**) pressure on evaporation rate of a mixture of 50% *n*-heptane and 50% *n*-hexadecane in liquid phase and at six different mole fractions in the gas phase.

We distinguish the quasi-equilibrium phenomena induced by the “internal rotations” dynamics relevant to thermal evaporation effects over the interfacial layers from equilibrium mass evaporation/condensation occurring between the gas and liquid phases. For the sake of simplicity, the model used in Fig. 3 includes two active site loops for two-component systems. Although, in reality there may be a large number of different conformers R_i_ and P_i_ (i = 1,2,3,….), we examine nano-confinement mechanistic hypothesis in which two conformers of each component are confined across the interface to be actively involved in phase transitions (see Fig. 3). We will also only consider the case of an ideal liquid mixture with incompressible liquid components and an ideal vapour mixture with each vapour component treated as an ideal gas. The expressions for Gibbs free energy of each component and their mixtures in liquid and vapour phases are given by:^43^

7 $${G}_{g}=\sum _{ci}{x}^{{C}_{i}}{G}_{g\,}^{{C}_{i}}+{k}_{B}T\sum _{ci}{x}^{{C}_{i}}ln{x}^{{C}_{i}}$$

8 $${G}_{l}=\sum _{ci}{x}^{{C}_{i}}{G}_{l\,}^{{C}_{i}}+{k}_{B}T\sum _{ci}{x}^{{C}_{i}}ln{x}^{{C}_{i}}$$

9 $${\rm{\Delta }}{G}_{g}^{mix}=+{k}_{B}T\sum _{ci}{x}^{{C}_{i}}ln{x}^{{C}_{i}}$$

10 $${\rm{\Delta }}{G}_{l}^{mix}=+{k}_{B}T\sum _{ci}{y}^{{C}_{i}}ln{y}^{{C}_{i}}$$

where y^Ci^ and x^Ci^ refer to mole fractions of i^th^ component C in the liquid and vapour phases, respectively. In this study these components are C_7_ and C_16_. Substituting equations (7–10) in KGT-based equation (6), we obtain the final expression for predicting evaporation flux for each component in a c-component mixture. Equation 3 can also be rearranged to predict evaporation rates for multicomponent liquid mixtures. Although details of the model presented here are novel for better understanding of new mechanistic pathways in evaporation of multicomponent fuel droplets, we note that it shares similarities with previous models applied for kinetics modeling of mono-component hydrocarbon droplets evaporation^9, 14^. We do not claim that QTST/IKGT based on this nano-confinement hypothesis should be taken as the correct kinetic model for each hydrocarbon. Nevertheless, the results in Fig. 4a,b show that QTST/IKGT establish temperature and pressure dependence of the evaporation rate of binary fuels, as long as two “equilibrium” conformational changes in liquid and gas phases are cyclically switched on and off with two other “quasi-equilibrium” transition states at the liquid-gas interface of each component (those are controlled by k_1_ (k′_1_) and k_2_ (k′_2_) – see Fig. 3). We also note that neglecting conformational effects in hydrocarbons in which evaporation rate is treated with a temperature-dependent term has been proposed by Elliott and War^18, 23^. We used equation (3) to fit the experimental evaporation rate of binary fuel of *n*-heptane and *n*-hexadecane hydrocarbons reported by Ghassemi and co-workers^44^ at different pressures and temperatures. The A_jk_ during the evaporation are constrained based on the experimental results^44^ which change from 5.181 to 0.471 mm^2^ at pressures 0.1 and 2.0 MPa. We then constrained transition-state frequencies, to lie between 100 and 1,100 cm^−1^, which are consequences of internal rotations in *n*-C_7_H_16_ and *n*-C_16_H_34_ at the interface. With these constraints over the temperature and pressure ranges 670–970 K and 0.1–2.0 MPa, we obtained the physically reasonable parameters given in Table 2 and results in Fig. 4 for two coupled conformers of each component in each phase. Indeed the hybrid QTST/IKGT method is inspired by discrete methods such as the Lattice Boltzmann methodology^45–49^ and *Lattice Boltzmann simulations* ^50–52^. The current hybrid methodology is explained by jiggling and wiggling of atoms in a few discretised conformers in the very vicinity of fuel droplets surfaces (both in pure^14^ and binary fuels). Their energies have been very well quantized and there is therefore no continuity (see Table 2). While the conversions of these conformers in the gas and liquid phases are taking place easily, their conformational changes at the interface need to pass specific pathways which are switched on-off. The conformational changes in conformers, collision rate effects and equilibrium vapour concentrations of the components in the gas phase play key roles to make ready these nano-pathways for the dynamic coupling between gas and liquid phases which are really important in the phase transitions (e.g. evaporation). Following from Fig. 4a, the model provides the results (red and green solid lines) which are fitted very well with the experimental evaporation rate (shown with star and cub symbols) respect to the temperatures 670–970 K once the X^C16^ and X^C7^ are respectively equal to 0.08 and 0.04 at pressure 0.5 MPa and 0.04 and 0.005 at pressure 0.1 MPa. Deviations are significant when these equilibrium vapour concentrations are a little change. The same scenario took place when the pressure dependency of evaporation rates of C7 and C16 was studied (see Fig. 4b).

**Table 2:** Parameters* obtained from fitting the data in Fig. 3a and b.

Binary Fuel	*C*_*7*_ *(C*_*16*_ )**		*C*_*7*_ *(C*_*16*_ )***		*C*_*7*_ *(C*_*16*_ )****		*C*_*7*_ *(C*_*16*_ )*****	
Parameters/interfacial conformer i	1	2	1	2	1	2	1	2
${G}_{i}^{{[{R}_{i}-{P}_{i}]}^{\#}}$	22.2 (18.1)	23.8 (21.2)	23.8 (17.8)	21.3 (19.7)	16.9 (18.1)	18.1 (16.7)	16.1 (14.5)	17.4 (16.2)
${U}_{i}^{{[{R}_{i}-{P}_{i}]}^{\#}}$	19.3 (17.6)	25.6 (18.9)	27.8 (19.6)	22.9 (20.9)	17.8 (16.7)	10.1 (17.3)	17.3 (19.1)	26.1 (23.8)
ω_i_	786 (985)	973 (1021)	898 (935)	932 (1025)	563 (764)	873 (761)	623 (845)	983 (1045)

*Units are as follows: ${G}_{i}^{{[{R}_{i}-{P}_{i}]}^{\#}}$ and ${U}_{i}^{{[{R}_{i}-{P}_{i}]}^{\#}}$ (kcal mol^−1^) and *ω*_i_ (cm^−1^).

**C_7_ (C_16_) refers to *n*-heptane (*n*-hexadecane) molecules. The parameters obtained at pressure 0.5 MPa and vapour mole fraction X^C7^ and X^C16^ are respectively 0.04 and 0.08.

***The parameters obtained at pressure 0.1 MPa and vapour mole fraction X^C7^ and X^C16^ are respectively 0.005 and 0.03.

****The parameters obtained at temperature 773 K and vapour mole fraction X^C7^ and X^C16^ are respectively 0.04 and 0.08.

*****The parameters obtained at temperature 673 K and vapour mole fraction X^C7^ and X^C16^ are respectively 0.005 and 0.03.

## Conclusion

In summary, the results of QTST/IKGT which have been inspired by the “*discrete*” Boltzmann method provide a new important physical concept for understanding dynamic coupling between liquid and gas phases during evaporation of multicomponent fuel droplets. This QTST/IKGT level of thermal and mass transport description in the vicinity of evaporating and condensing fuel droplets indicates two concerted-distinct hydrocarbon topologies in each component for coupling thermal – mass evaporation upon phase transitions. Moreover, we also gain further physical insight into the pathways followed by switching on and off mechanisms at the interface via internal rotations – this insight was previously lacking for multicomponent systems. These pathways are very sensitive to the collision effects, and conformational changes and equilibrium vapour concentrations next to interface. Moreover, the approach presented herein is anticipated to lead to a more refined QTST/IKGT method for reactive multicomponent interfacial transport as simple adsorption – desorption of long chain molecules on a substrate can induce not only conformational changes, but also spontaneous breaking of covalent carbon – carbon bonds^53^.

## Methods

### Thermal Evaporation Coefficient and Reactive MD simulations

**Figure 5:**
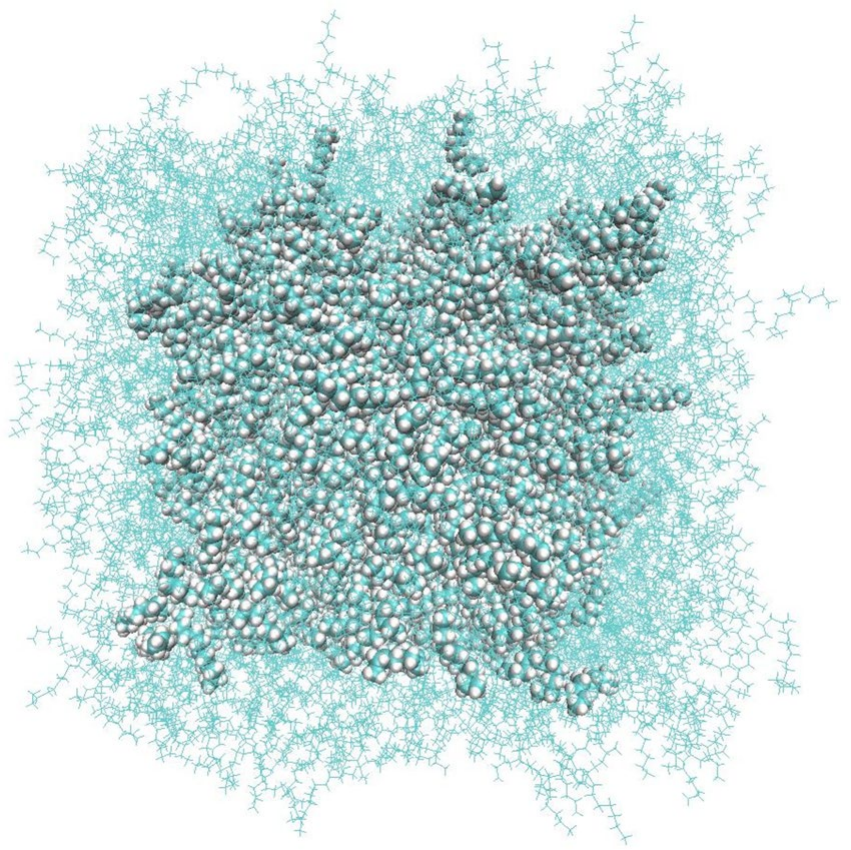
A schematic view of a nano-droplet. The droplet has a diameter of 10 nm (96900 atoms); the liquid phase is surrounded by the interfacial layer of thickness of about 1.7 nm when the system is heated up to 400 K. The location of the Gibbs dividing surface that corresponds to the area where the density is equal to 0.5 (ρ_liq_ + ρ_vap_) is used to estimate thickness of interfacial layer (see equation (19) for more detail).

In our approach the droplet (see Fig. 5) was first minimised and subsequently pre-equilibrated to desired temperatures of 350 and 400 K. The Berendsen’s thermostat^40^ controlled the kinetic energy of the system by scaling the velocities. A Velocity-Verlet algorithm was used to integrate the equations of motion. After equilibrating the systems, the interface layers were strongly coupled with thermostat (with relaxation time τ_T_ = 1 fs) while the rest of the system was weakly coupled with τ_T_ = 100 ps. The “coupling time constant”, τ, was used to estimate the time evolution of temperatures based on this equation:

11 $$\frac{dT}{dt}={\tau }^{-1}[{T}_{0}-T(t)]$$

where τ = 2τ_T_ *C*_*V*_/(*N*_*f*_ *k*_*B*_), *C*_*V*_ is the specific heat capacity at constant volume, *k*_*B*_ represents the Boltzmann constant, and *N*_*f*_ is the number of degrees of freedom of the system. The time constants, by which systems are allowed to reach the quasi-equilibrium state in micro-canonical conditions (NVE), clarified the β_T_, for which energy transformations were considered via the interface in a non-steady way and exchanged suddenly. This method allowed us to study gradients of temperature during the evaporation/condensation processes in the vicinity of the liquid-gas interface. We used the Amsterdam Density Functional (ADF) package^54^ for all ReaxFF simulations.

**Figure 6:**
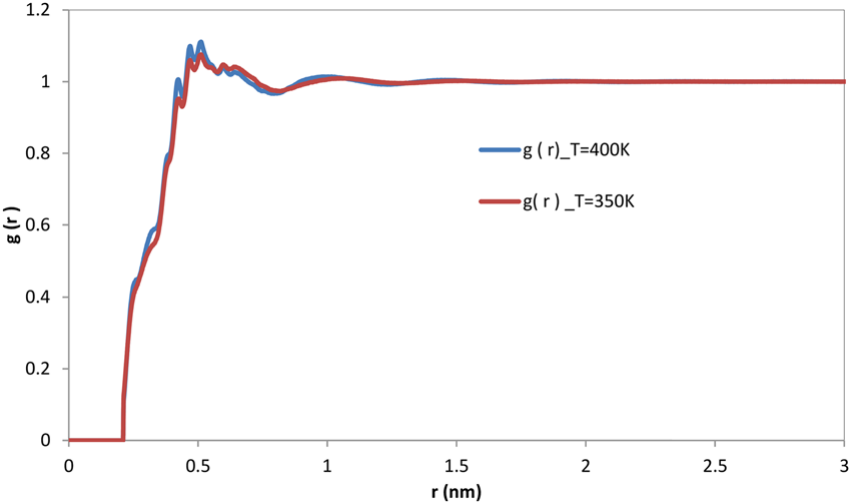
Pair correlation function for *n*-dodecane at 350 K and 400 K as function of r (distance of centre of mass of the molecules).

The temperature in the system under consideration is estimated based on the analysis of various parts of the system (*e.g*. interface, gas, liquid, drop) separately. The analysis of the interface has been performed only for the molecules which stay in the drop during the whole simulation, ignoring the molecules which leave the droplet. The average energy of gas molecules was obtained based on gas (vapour) temperature which was determined from the conservation of energy:

12 $${T}_{{\rm{g}}}=\frac{{T}_{{\rm{total}}}{N}_{{\rm{total}}}-{T}_{{\rm{drop}}}{N}_{{\rm{drop}}}}{{N}_{{\rm{g}}}},$$

where subscript ‘drop’ refers to the sum of the interfacial layer and liquid phase as shown in Fig. 5. The number of evaporated molecules (*N*_g_) was estimated based on a cut-off distance at which molecules belong to the drop or to the gas phase. It was set to 0.5 nm as inferred from the pair correlation function (*g*(*r*)) of *n*-dodecane (see Fig. 6).

### Mass Evaporation Coefficient and Quantum/Statistical Mechanics Methods

We have first estimated interfacial width, δ, which was unknown in the equation (2), using the following equation:^55–58^

13 $$\mathrm{ln}\,\frac{\delta }{\sigma }=\,\mathrm{ln}\,[a{(1-\frac{T}{{T}_{c}})}^{-\upsilon }]$$

where σ is a temperature-independent diameter parameter of the methylene and methyl functional groups in *n*-dodecane conformers, which is assumed to be 3.93 × 10^−10^ m; a = 1.16 m and υ = 0.5 are constants and *T*_c_ = 658.15 K is the critical temperature for *n*-dodecane. We apply the multistructural statistical thermodynamic method^59^ alongside density functional theory to calculate the Gibbs free energies of *n*-Dodecane conformers in the gas phase (*G*_*g*_(*T*));^60^

14 $${G}_{g}(T)=-RT\,\mathrm{ln}({Q}_{g}^{MS-T})+RT,$$

where ${Q}_{g}^{MS-T}$ represents the multi-structural partition functions in the gas phase in which rotational, vibration, conformational and torsional effects have been taken into account based on the following formulae:

15 $${Q}_{g}^{MS-T}=\sum _{i=1}^{N}{Q}_{rot,i}{Q}_{vib,i}\exp (-\frac{{U}_{i}}{{k}_{B}T})\times \prod _{\tau =1}^{t}{\varphi }_{i,\tau },$$

where *k*_B_ is the Boltzmann constant, and *U*_*i*_ is the energy of the *i* ^th^ conformer, *N* is the number of conformers and *ϕ*_*i*,*τ*_ is a factor that takes account of torsional potential anharmonicity. Q_rot,i_ is a classical expression for the rotational partition function for conformer i;

16 $${Q}_{rot,i}=\frac{\sqrt{\pi }}{{\sigma }_{rot,i}}(\frac{2{k}_{B}T}{{\hslash }^{2}})\sqrt{{I}_{A,i}{I}_{B,i}{I}_{C,i}},$$

where *σ*_*rot*_,_*i*_ ≥ 1 is the symmetry number of the molecule, and *I*_*A*,*i*_ *, I*_*B*,*i*_, and *I*_*C*,*i*_ are principal moments of inertia. *Q*_*vib,i*_ is the usual harmonic oscillator vibrational partition function calculated at conformer i using the following expression;

17 $${Q}_{vib,i}=\prod _{i=1}^{F}\frac{\exp (-\frac{\hslash {\omega }_{i,l}}{2{k}_{B}T})}{1-\exp (-\frac{\hslash {\omega }_{i,l}}{{k}_{B}T})}$$

where *F* and *ω*_*i*,*l*_ indicate the number of degrees of freedom for vibration modes and vibration frequency of the l^th^ mode of the i^th^ conformer, respectively. To calculate the Gibbs free energies of each conformer at the interface (*G*_*i*_(*T*)) we employ a modified version of continuum solvation model SMD^35, 61^ in which some correction terms in temperature dependence of interfacial density and surface tension have been taken into account. SMD is based on the solute electron density, the dielectric constant and the atomic surface tension. The temperature dependence of the surface tension is included using the following formula:^62^

18 $${\rm{\Omega }}=B{(1-\frac{T}{{T}_{c}})}^{n},$$

where *B* and *n* are constants: *B* = 80.1946*10^−3^ kcal/(mol*Å^2^), *n* = 1.3325, and *T*_*c*_ is the critical temperature of *n*-dodecane. The temperature dependency of interfacial density of *n*-dodecane is also computed with the self-consistent reaction field (SCRF) method, implemented in the Gaussian 09 suite^63^. The interfacial density, *ρ(z)*, can be expressed as a hyperbolic tangent function:

19 $$\rho (z)=\frac{1}{2}({\rho }^{l}+{\rho }^{g})-\frac{1}{2}({\rho }^{l}-{\rho }^{g})\tanh [\frac{2(z-{z}_{0})}{\delta }],$$

where superscripts l and g denote liquid and gas phases, respectively, and z_0_ is the position of the Gibbs dividing surface. The saturated densities of liquid and gas at temperatures T = 298.15 K to 648.15 K are taken from the NIST^64^. Since the translational motions are suppressed at the surface of liquid and all SMD calculations have also been performed based on existence of a conformer in the cavity, the pressure corrections also need to be taken into account using;

20 $$p=\rho RT[1+\delta {(\frac{\partial A}{\partial \delta })}_{\tau }],$$

where *p* is the pressure, τ = *T*_c_/*T*, *δ* = *ρ*/*ρ*_c_, *ρ* and *ρ*_c_ = 1.33 mol/dm^3^ are the density and critical density of *n*-dodecane, respectively, and *A* is the Helmholtz free energy:^65^

21 $$\begin{array}{rcl}A(\delta ,\tau ) & = & \delta ({n}_{1}{\tau }^{0.32}+{n}_{2}{\tau }^{1.23}+{n}_{3}{\tau }^{1.5})\\  &  & +{\delta }^{2}[{n}_{4}{\tau }^{1.4}+{n}_{5}\delta {\tau }^{0.07}+{n}_{6}{\delta }^{5}{\tau }^{0.8}+{n}_{7}{\tau }^{2.16}\exp (-\delta )]\\  &  & +{n}_{8}{\delta }^{5}{\tau }^{1.1}\exp (-\delta )+\delta \exp (-{\delta }^{2})({n}_{9}{\tau }^{4.1}+{n}_{10}{\delta }^{3}{\tau }^{5.6})\\  &  & +{\delta }^{3}\exp (-{\delta }^{3})({n}_{11}{\tau }^{14.5}+{n}_{12}\delta {\tau }^{12})\end{array},$$

where p is the pressure in the centre of interfacial layer and *ρ* is the experimental interfacial density of *n*-dodecane changing from 372.8 kg/m^3^ at 298.15 K to 117.5 kg/m^3^ at 648.15 K^64^. The constants *n*_1_, *n*_2_,…*n*_12_ are given in Table 3.

**Table 3:** Constants for the Helmholtz free energy of *n*-dodecane at the interfacial layer^65^.

Parameter	Value	Parameter	Value
*n*_1_	1.38031	*n*_7_	0.956627
*n*_2_	−2.85352	*n*_8_	0.0353076
*n*_3_	0.288897	*n*_9_	−0.445008
*n*_4_	−0.165993	*n*_10_	−0.118911
*n*_5_	0.0923993	*n*_11_	−0.0366475
*n*_6_	0.000282772	*n*_12_	0.0184223

The Gibbs free energy of the ensemble of conformers at the interface was determined by the formula;

22 $${G}_{int}=-RTln\sum _{i=1}^{N}\exp (-{G}_{i}/{{\rm{k}}}_{{\rm{B}}}{\rm{T}}),$$

This equation was applied to conformer ensembles in both gas and liquid phases. The average changes in the Gibbs free energy upon evaporation (or condensation) of a molecule in the equation (2) (<Δ*G*_g↔int_>) were estimated as;^9^

23 $$\langle {\rm{\Delta }}{{G}}_{g\leftrightarrow \mathrm{int}}\rangle =({{\rm{G}}}_{{\rm{int}}}-{{\rm{G}}}_{{\rm{g}}})/{\rm{N}}$$

where subscripts _int_ and _g_ refer to the interface and gas phase.
